# Validation of the two-minute step test in obese with comorbibities and morbidly obese patients

**DOI:** 10.1590/1414-431X20198402

**Published:** 2019-08-29

**Authors:** P.A. Ricci, R. Cabiddu, S.P. Jürgensen, L.D. André, C.R. Oliveira, L. Di Thommazo-Luporini, F.P. Ortega, A. Borghi-Silva

**Affiliations:** 1 Laboratório de Fisioterapia Cardiopulmonar, Universidade Federal de São Carlos, São Carlos, SP, Brasil; 2 Departamento de Medicina, Universidade Federal de São Carlos, São Carlos, SP, Brasil; 3 Santa Casa de Misericórdia de São Carlos, São Carlos, SP, Brasil

**Keywords:** Cardiopulmonary exercise test, Oxygen uptake, Obesity, Cardiorespiratory fitness

## Abstract

Cardiopulmonary fitness assessment is a valuable resource to obtain quantitative indicators of an individual's physical performance. The cardiopulmonary exercise test (CPX), considered the gold standard test for this evaluation, is costly and difficult to be accessed by the general population. In order to make this evaluation more accessible, and to better reflect the performance of daily life activities, alternative tests were proposed. Morbidly obese patients present limitations that impair physical performance assessment and could benefit from a test of shorter duration, provided it is validated. This observational study aimed to validate the two-minute step test (2MST) as a tool to evaluate functional capacity (FC) in obese with comorbidities and morbidly obese patients, compared the 2MST with CPX as a measure of physical performance, and developed a predictive equation to estimate peak oxygen uptake (VO_2_) in the 2MST. The CPX and the 2MST were performed and metabolic and ventilatory parameters were recorded in 31 obese individuals (BMI>35 kg/m^2^). Pearson correlation and multiple linear regression analyses were performed to evaluate the peak VO_2_ best predictors. Bland-Altman analysis was performed to assess the agreement between the two methods. Peak VO_2_ measured by CPX and 2MST showed a strong correlation (r=0.70, P<0.001) and there was a moderate correlation between peak VO_2_ of the 2MST and the number of up-and-down step cycles (UDS) (r=0.55; P=0.01). The reference equation obtained was: VO_2_ (mL·kg^-1^·min^-1^) = 13.341 + 0.138 × total UDS – (0.183 × BMI), with an estimated standard error of 1.3 mL·kg^-1^·min^-1^. The 2MST is a viable, practical, and easily accessible test for FC. UDS and BMI can predict peak VO_2_ satisfactorily.

## Introduction

Cardiopulmonary fitness assessment is a valuable resource to obtain quantitative indicators of an individual's physical performance. The results of this evaluation are fundamental for the elaboration of physical exercise prescriptions (1), especially for populations that need this as the first line of treatment, such as obese patients or patients with chronic diseases directly related to functional capacity (FC) impairment.

The cardiopulmonary exercise test (CPX) is the gold standard test (2,3) for this evaluation and a valuable tool for diagnosis elaboration. However, several factors make this evaluation costly, thus limiting access by the general population. Among others, disadvantages include the need for adequate space for the use of the ergometer, as well as the necessity of trained professionals to apply the test and interpret the results. In addition, the CPX presents a maximum characteristic (4) and determines a greater chronotropic stress with higher peak oxygen uptake (VO_2_), which leads to greater fatigue compared to field tests.

In order to make this evaluation more accessible, minimize costs, and better reflect the performance of daily living activities (5), other tests have been proposed for the evaluation of FC. Morbidly obese patients have limitations, such as hypertension, diabetes mellitus, and arthropathies (6) that limit cardiopulmonary evaluation through CPX. The 6-min step test (6MST) has already been applied at submaximal and near-maximal intensities to assess FC and even predict cardiorespiratory fitness (VO_2_) in obese patients (1,5). However, the duration of the test can be very exhaustive for some patients, leading to discontinuation due to muscle fatigue symptoms and dyspnea (5).

Rikli and Jones (7) showed that the two-minute step test (2MST) can be reliably used in healthy elderly individuals. Wegrzynowska-Teodorczyk et al. (8) found that the 2MST can be considered reliable to assess FC in heart failure patients. An alternative would be the performance of a step test with the same duration (2 min) on a step ergometer, which requires the patient's vertical and horizontal displacement, and not a march-in-place test, as both studies performed.

To date, no study assessed the 2MST validity as a method to assess FC in morbidly obese individuals; however, this population could benefit from a shorter duration test, provided it is validated.

Thus, the objectives of the present study were: 1) to verify the validity of the 2MST as a tool to evaluate FC in obese with comorbidities and morbidly obese patients; 2) to compare the 2MST with CPX as a measure of physical performance; and 3) to develop a predictive equation to estimate peak VO_2_ with the 2MST.

## Material and Methods

### Study design

The present study was done at the Physiotherapy Department of the Federal University of Sao Carlos (UFSCar), in the Cardiopulmonary Physiotherapy Laboratory (LACAP), from March 2016 to December 2017. Ninety-one patients between 18 and 60 years old, candidates for bariatric surgery, were recruited by means of advertisements and medical indications, however, only 31 concluded the study.

This observational study was conducted according to the Consensus-based Standards for the Selection of Health Status Measurement Instruments (COSMIN) (9). Participants were informed about study objectives, procedures, and risks. All participants signed an informed consent form. The study was approved by the UFSCar Ethics and Research Committee (No.966.613).

### Inclusion and non-inclusion criteria

Obese with comorbidities (body mass index (BMI)≥35 kg/m^2^) and morbidly obese individuals (BMI≥40 kg/m^2^), with sedentary lifestyle, were included in the study. The criteria for a sedentary lifestyle were a maximum of 150 min of physical activity per week and Baecke questionnaire score below 8 (10).

Non-inclusion criteria considered the presence of: orthopedic or neurological impairment; myocardial infarction; implanted pacemaker or any metallic prosthesis; unstable angina; chronic heart rhythm disorders; moderate or severe heart valvular disease; uncontrolled hypertension; uncontrolled and/or insulin-dependent diabetes mellitus; use of beta-blockers; participation in a regular exercise program at the beginning of the study; respiratory diseases; any contraindication to the ergospirometry test; conditions that could compromise the performance of functional tests; distal arteriopathies; inflammatory, renal or hepatic conditions; documented diabetic neuropathy; cognitive deficits; declared use of illicit drugs and gestation.

### Experimental protocol

The experimental protocol consisted of two visits. In the first visit, a clinical evaluation was performed by anamnesis and anthropometry, followed by the CPX. All evaluations were performed in an air-conditioned environment and occurred at the same time of the day. Participants were instructed not to consume caffeine, alcoholic beverages, or any other stimulants the night before and the day of data collection, nor to perform strenuous activities the day before the visits.

The anthropometric test was performed using a stadiometer (Welmy R-110, Brazil) to measure height and body mass. Volunteers were instructed to stand barefoot and to wear light clothes. Body composition analysis was performed using a digital scale (Model InBody 720, Biospace, Korea). Volunteers performed this evaluation in the morning. They were instructed to have an absolute fast of at least four hours and to eliminate urine prior to the evaluation. Lean muscle mass (kg) and fat mass (kg) values were obtained.

The Baecke physical activity questionnaire, validated for Brazilian adults (11), was applied. Afterwards, the participants were invited to familiarize themselves with the ergometer (Super Inbramed ATL treadmill, Brazil) that would be used to perform the CPX. Before starting the CPX, the volunteer remained in seated rest for two minutes, followed by two minutes of standing. A CPX with a maximum characteristic and/or symptom-limited was performed according to the Bruce protocol (12). This protocol presents progressive increases in speed and inclination every three minutes. Upon reaching the peak of exercise, the protocol was interrupted, the inclination was reset and the speed maintained at three miles per hour for an active recovery period of 3 min. After this period, passive recovery was started, with the volunteer sitting for 3 min.

Throughout the CPX, metabolic and ventilatory parameters were recorded breath by breath by a portable Oxycon Mobile^®^ ergospirometry system (Mijnhardt/Jäger, Germany). The system was calibrated before each test. A face mask was used by the participants as an interface between expired gases and equipment. For the evaluation of VO_2_, data obtained at the peak of exercise were used. Data were processed and 15 s moving average values were obtained. The highest value of the last 15 s was defined as the peak value of VO_2_. Carbon dioxide production (VCO_2_), respiratory exchange ratio (RER), minute ventilation (VE), respiratory rate (RR), and the VE/VCO_2_ were measured and exported to Excel^®^ (Microsoft Excel, 2016). Any missing values due to technical equipment problems were treated as missing data and not considered for the final analysis.

Blood pressure (BP) was measured during rest, every 3 min during the test, at the peak of the exercise, and at the end of active and passive recovery by a sphygmomanometer (Becton Dickinson, Brazil) and a stethoscope, by the auscultatory method. Heart rate (HR) was monitored by means of a heart rate monitor (Polar^®^ S810i, Finland) throughout the test. Subjective dyspnea and lower limb fatigue responses were collected through the Borg visual scale (0 to 10) (13).

The CPX was conducted by a physician and two properly trained physiotherapists; for safety purposes, all volunteers were continuously monitored by a 12-lead electrocardiogram (ECG) (Wincardio, Micromed, Brazil). Volunteers were encouraged to perform the test until exhaustion and the criteria for interruption/completion of the test followed the American Thoracic Society recommendations (2).

The second visit occurred after a minimum interval of 48 h after the first one, in which the individuals performed the 2MST. After familiarization with the ergometer, volunteers were instructed to go up and down a portable, rubberized, non-slippery 15 cm step, with no hand support, as many times as possible for 2 min, with free cadence, and being able to slow down, speed up, or even interrupt the exercise if necessary. An evaluator was responsible for counting up-and-down step cycles (UDS). The proper cycle was demonstrated to the patient; the cycle should be initiated with the ascent on the step with the dominant limb, followed by the contralateral limb and the descent should be carried out in the same order.

Before the test, volunteers performed 2 min of rest in a seated position, followed by 2 min in orthostatism. Afterwards, the exercise test started, with standardized verbal encouragement after the 1st min. At the end of the exercise, subjects remained seated for 6 min (passive recovery).

The workload (in W) performed during the test was calculated as: step height (m) × total UDS × weight (kg) × 0.16357 (14). Throughout the test, measurements of expired gases and HR were collected, according to the same methodology described for the CPX. BP and the subjective sensation of fatigue on the Borg scale were recorded during the initial rest, at the end of the exercise, and at the 3rd and 6th min of passive recovery. At the end of the test, the number of UDS was considered. Two properly trained physiotherapists were responsible for conducting the test.

### Data analysis

ECG data recorded during the CPX were stored in the WinCardio software (Micromed) for further analysis. Metabolic and ventilatory data were processed and calculated in rolling averages every 15 s. During the rest period, the mean value of the 1st min was considered for all the metabolic and ventilator parameters.

The HR data were stored in a clock and downloaded through an infrared interface to the Polar Pro Trainer 5 software (Polar^®^ S810i) on a computer, and subsequently tabulated for future analysis.

BP values, the subjective perception of fatigue by the Borg scale, and the performance during the 2MST (number of UDS cycles performed) by each individual were recorded manually in the evaluation form of each patient and later tabulated.

### Statistical analysis

Statistical analysis was performed using SigmaPlot, version 11.0. Data are reported as mean and 95% of confidence interval. Data normality was tested by the Shapiro-Wilk test. An *a posteriori* power analysis was performed using the GPower statistical package, Version 3.1.3 (Franz Faul Universität Kiel, Germany). Considering our study total sample size of 31 individuals, 5% type I error, an effect size of 0.50, and the total number of predictors of 2, the statistical power was calculated to be 93%.

Student's *t*-test or Wilcoxon test were used to compare 2MST and CPX variables. Pearson's correlation coefficient was used to study the correlations between variables and evaluate the degree of association between CPX and 2MST measurements. The r values were interpreted using the following guidelines: 0.00 to 0.19=none to slight, 0.20 to 0.39=low, 0.40 to 0.69=modest, 0.70 to 0.89=high, and 0.90 to 1.00=very high (15). Bland-Altman (16) analysis was performed to evaluate the agreement between VO_2_ values in both tests. Stepwise multiple linear regression analysis was performed to evaluate the best predictors of CPX and 2MST peak VO_2_. The reliability of the reference equation for prediction of the 2MST peak VO_2_ was tested on a second group composed by 10 individuals; to this aim, the real measurements obtained by these subjects in the 2MST and the values obtained from the equation were compared. The level of statistical significance was set at 5%.

## Results

**Figure 1.**
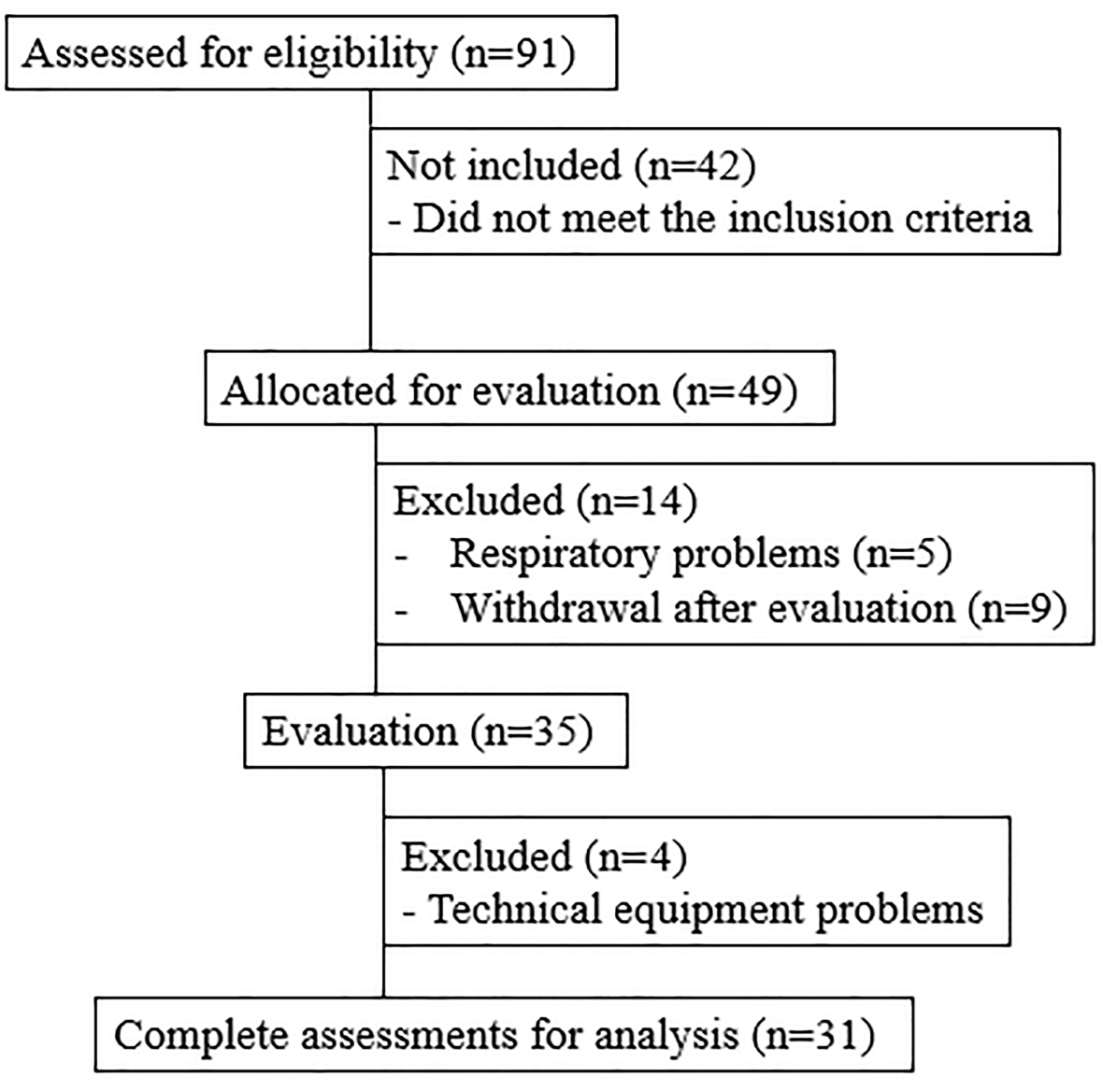
Flowchart of the individuals who participated in the study.

Figure 1 illustrates the recruitment flowchart of the individuals who participated in the study. We recruited 91 obese individuals, and the final sample was composed by 31 volunteers. The volunteers' general characteristics are presented in Table 1.

**Table 1:** General characteristics of the studied population.

Variables	n = 31 (95% CI)
Age, years	37.3 (33.7 to 41.0)
Gender, %	M: 19.2%; W: 80.8%
Weight, kg	115.9 (109.6 to 122.3)
Height, cm	166.0 (163.1 to 169.9)
BMI, kg/m^2^	41.7 (39.9 to 43.4)
Fat Mass, kg	57.2 (47.0 to 50.8)
Lean Mass, kg	55.9 (52.6 to 59.1)
Physical activity level (Baecke questionnaire)	6.8 (6.3 to 7.3)
Arterial hypertension, n (%)	23 (74.1%)
Diuretic, n (%)	4 (12.9%)
ARB, n (%)	8 (25.8%)
Diabetes mellitus, n (%)	5 (16.1%)
Metformin, n (%)	5 (16.1%)

Data are reported as mean and 95% CI (confidence interval) or number of patients (%). M: men; W: women; BMI: body mass index; ARB: angiotensin receptor blocker.

Table 2 shows the variables related to the metabolic and cardiovascular responses and to perceived exertion stress at the peak of the CPX and of the 2MST. The distance covered during the CPX was 474.1±127.8 m and the average number of UDS during the 2MST was 49.6±7.1. The workload during the 2MST was 141±31 W.

**Table 2:** Variables at the peak of the cardiopulmonary exercise test (CPX) and of the two-minute step test (2MST).

Variables	CPX (95% CI)	2MST (95% CI)	P value
Duration, min	7.7 (7.2 to 8.2)		
Metabolic data			
VO_2_, mL/min	1813.9 (1691.8 to 1935.9)	1468.0 (1368.5 to 1567.6)*	<0.001
VO_2_, mL·kg^-1^·min^-1^	15.6 (14.9 to 16.4)	12.5 (11.8 to 13.2)*	<0.001
VO_2_, % pred	81.8 (78.0 to 85.5)	65.9 (62.4 to 69.4)*	<0.001
VCO_2_, mL/min	2353.0 (2192.6 to 2513.4)	1436.7 (1326.9 to 1546.5)*	<0.001
RER	1.29 (1.2 to 1.3)	1.01 (0.96 to 1.06)*	<0.001
Ventilatory data			
V_E_, L/min	83.7 (78.6 to 88.9)	47.6 (43.5 to 51.8)*	<0.001
RR, br/min	43.6 (40.3 to 46.9)	30.5 (27.9 to 33.0)*	<0.001
V_E_/VCO_2_	28.3 (26.8 to 29.7)	30.8 (29.0 to 32.6)*	0.005
Cardiovascular data			
Heart rate, bpm	170.0 (164.6 to 175.5)	135.8 (129.4 to 142.3)*	<0.001
% HRmax	93.3 (90.4 to 96.3)	74.3 (70.9 to 77.7)*	<0.001
SBP, mmHg	194.8 (185.3 to 204.4)	181.3 (168.6 to 193.9)	0.108
DBP, mmHg	102.3 (98.2 to 106.5)	102.0 (97.5 to 106.6)	0.691
DP	33,144.0 (31,271.6 to 35,016.3)	24,708.6 (22,417.1 to 27,000.0)*	<0.001
Symptoms (Borg scale)			
Dyspnea	7 (3;10) (6.8 to 8.4)	2 (0;7) (1.6 to 3.3)*	<0.001
Leg fatigue	3 (0;10) (2.7 to 5.1)	0 (0;6) (0.5 to 1.9)*	<0.001

Data are reported as mean or median, according to data distribution, and CI (confidence interval). CPX: cardiopulmonary exercise test; 2MST: two-minute step test; VO_2_: oxygen uptake; VCO_2_: carbon dioxide production; RER: respiratory exchange rate; V_E_: minute ventilation; RR: respiratory rate; br: breaths HR: heart rate; HRmax: maximal heart rate; SBP: systolic blood pressure; DBP: diastolic blood pressure; DP: double product. *Significant differences between CPX and 2MST. Student's *t*-test or Wilcoxon test were used for statistical analysis.

**Figure 2.**
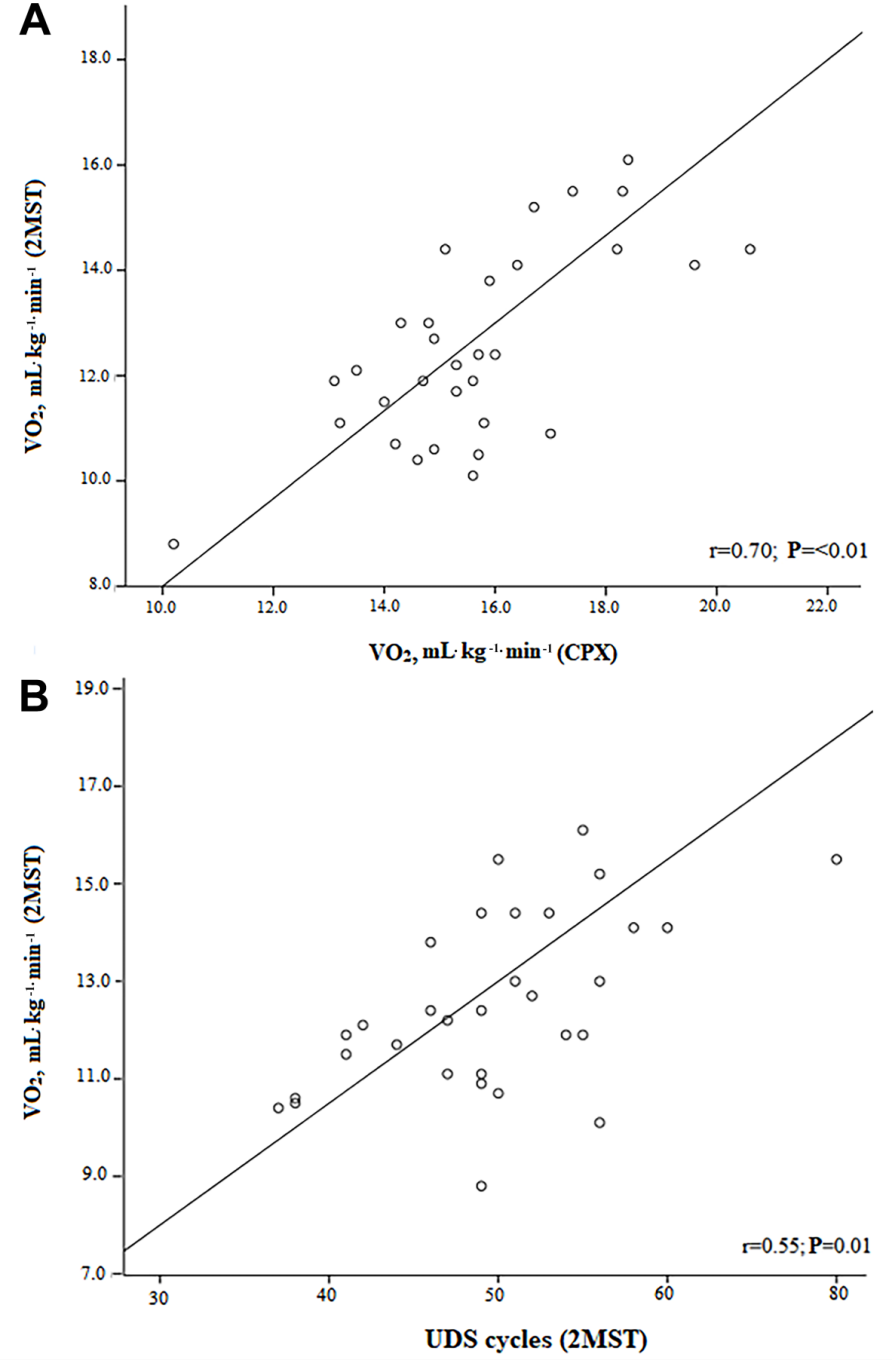
**A**, Correlation between oxygen uptake (VO_2_) at the peak of the cardiopulmonary exercise test (CPX) and at the peak of the two-minute step test (2MST). **B**, Correlation between VO_2_ at the peak of the 2MST and the up-and-down step cycles (UDS).

The correlation analysis showed that there was a strong correlation between VO_2_ at the peak of the CPX and at the peak of the 2MST (r=0.70; P<0.001). Moreover, a modest correlation was observed between VO_2_ at the peak of the 2MST and the UDS (r=0.55; P=0.01) (Figure 2).

**Figure 3.**
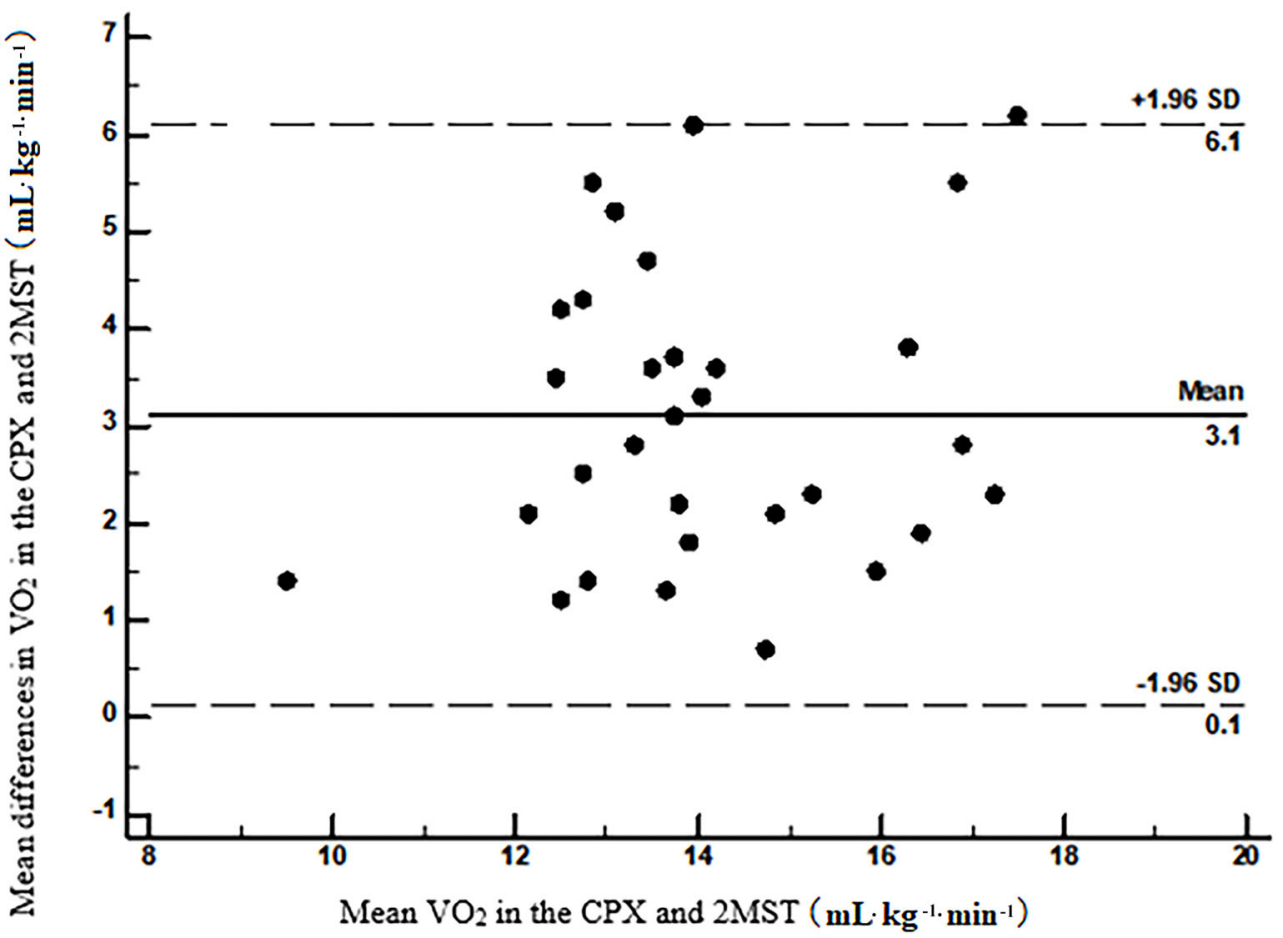
Bland-Altman graphical analysis of the differences between peak VO_2_ of the cardiopulmonary exercise test (CPX) and the two-minute step test (2MST).

Figure 3 shows the results of the Bland-Altman analysis; the mean values of the differences between peak VO_2_ of both tests were close to zero, suggesting there was agreement between the two methods.

A multiple linear regression analysis was performed, considering the variables BMI and number of UDS obtained during the 2MST, which explained 53% of the VO_2_ variance at the peak of the 2MST. Thus, the following reference equation was obtained: VO_2_ (mL·kg^-1^·min^-1^) = 13.341 + 0.138 × total UDS - (0.183 × BMI), with an estimated standard error of 1.3 mL·kg^-1^·min^-1^. Table 3 shows the variables related in the reference equation.

**Table 3:** Oxygen uptake (VO_2_) predictive model considering number of up-and-down step cycles (UDS) during the two-minute step test (2MST) and body mass index (BMI).

Variables	Coefficient	SE	P value
R^2^ = 0.534			
Constant	13.341	2.701	<0.001
UDS	0.138	0.033	<0.001
BMI	-0.183	0.050	<0.001

Ten participants were recruited to test the equation. Their characteristics were as follows: age=34.7±5.9 years; stature=1.64±0.07 m; weight=110.3±17.5 kg; BMI =40.6± 4.8 kg/m^2^; UDS=53.5±8.0. The same procedures were performed with these subjects and, after performing the 2MST, mean peak VO_2_ was 12.0±4.3 mL·kg^-1^·min^-1^. To verify the equation validity, the values of UDS and BMI from these ten individuals were substituted in the equation. The mean predicted VO_2_ was 13.0±1.0 mL·kg^-1^·min^-1^ for the 2MST, with an error of 1.0 mL·kg^-1^·min^-1^ and no difference between them (P=0.9).

## Discussion

To our knowledge, this is the first study to propose the 2MST on a step ergometer in obese with comorbidities and morbidly obese patients suggesting that it can be considered as a valid test for FC evaluation in this population. The main results can be summarized as follows: 1) there was an association between peak VO_2_ with both tests - CPX and the 2MST; 2) there was agreement between the metabolic response to 2MST and CPX, supporting the 2MST clinical validity as a FC assessment tool; and 3) it was possible to develop a predictive equation to estimate peak VO_2_ starting from the 2MST results.

The step protocol was first described as a form of evaluation at the beginning of the 20th century; since then, several protocols have been developed over time (17). Different from some studies that use the stationary gait (7,8), we chose to use a step because we observed that the physical activity performed during an exercise that requires vertical and horizontal body displacement requires large muscle groups to make greater efforts (18), which can generate greater fatigue in obese individuals, but also reflect the performance of daily activities. The 6MST is a valid and reliable alternative for the evaluation of several populations, including disabled population (1,5,19) and even healthy individuals (20). However, the duration of the time-limited exercise test could be a barrier to morbidly obese individuals. Di Thommazo-Luporini et al. (5) reported that four morbidly obese patients discontinued the exercise from the 3rd min of execution of the 6MST due to muscle fatigue and dyspnea.

Dal Corso et al. (21) suggested that shorter duration tests might be suitable and that a 2- or 3-min test would provide a reasonable estimate of exercise capacity. Moreover, after the 3rd min of the step test, volunteers reach their steady state (5). In our study, we observed that the 2MST was able to generate sufficient metabolic stress for functional cardiopulmonary evaluation of individuals with a high degree of obesity.

Field tests have a high clinical value, since they allow to evaluate the impact of rehabilitation measures (22). In obese individuals, especially those with higher degrees of obesity and/or associated comorbidities, some protocols may make this assessment quite exhaustive and limiting, both for the cardiopulmonary and musculoskeletal systems. Thus, new alternatives are suggested to reduce risk and to make this evaluation easier and more practical. In view of this, the 2MST could be an option, mainly for this population, as an evaluation method that optimizes the performance of specific populations. In addition to the previously mentioned advantages, this test can be performed anywhere and it does not require the constant presence of a doctor, as long as it has a trained physiotherapist and adequate monitoring for patient safety.

In our study, the volunteers presented a difference in VO_2_ of 3.1±1.9 mL·kg^-1^·min^-1^ between CPX and 2MST, and no volunteer interrupted the test. Di Thommazo-Luporini and coauthors (5), who evaluated obese women, found a VO_2_ mean difference between the CPX and 6MST of 5.1±3.6 mL·kg^-1^·min^-1^, and they considered a satisfactory validity between CPX and 6MST, even with some morbidly obese individuals having had to interrupt the test. Thus, we believe that a shorter test time can minimize interruptions during the proposed exercise in the studied population, as well as reduce the musculoskeletal stress, improving the performance of the individual and, possibly, better reflecting the functional aerobic capacity. In their study, volunteers reached approximately 90% of the predicted VO_2_ (near maximal functional test) at the 6MST, while our patients reached 64% of the same parameter at the 2MST, characterizing it as a submaximal functional test. In this sense, the time duration proposed in the 2MST seems to be adequate to evaluate impaired populations, such as obese with comorbidities and morbidly obese individuals. Although our volunteers presented lower dyspnea, lower limb fatigue, and lower VO_2_ in 2MST compared to CPX, blood pressure responses were similar in both tests and there was no change in ECG.

In addition, obese individuals may present functional impairment (6), including gait changes (23), postural and balance deficits (24,25), and fatigue, which limit exercise with long duration; therefore, safe and effective tests are needed for cardiorespiratory evaluation in these patients. Some studies have observed higher values of dyspnea and fatigue during maximal tests compared to a submaximal test (12,26), and in our study, volunteers also presented similar responses during CPX, with significantly higher dyspnea and fatigue in the legs. Moreover, when compared with other studies that also performed CPX in morbidly obese individuals (27,28), we observed that our volunteers presented a lower peak VO_2_ value, even with our volunteers performing a maximal test (RER: 1.29 (1.2 to 1.3); HR%max: 93.3 (90.4 to 96.3). Although both exercise tests presented different intensity workloads (CPX, maximal characteristic; 2MST, submaximal characteristic), we verified that the 2MST presented satisfactory validity to evaluate FC in the studied population.

Some authors have developed predictive equations from submaximal functional tests (29–31) for healthy and disabled individuals. From easily accessible variables, one from the anthropometric evaluation (BMI) and the other from the performance obtained in the 2MST (UDS), it was possible to elaborate a reference equation to predict the 2MST peak VO_2_. These predictors together explained 53% of the total variance of VO_2_ at the peak of the 2MST.

Many studies used the 2MST as a tool to evaluate FC in different populations (32–34). The 2MST could be implemented in primary care and in hospitals, making an evaluation accessible to obese individuals with comorbidities, facilitating the implementation of rehabilitation strategies.

The daily practice of physical exercises and nutritional counseling are fundamental pillars for the treatment of obesity (35). The increase in energy expenditure with an increase of physical exercises associated to a decrease in sedentary habits is determinant for weight loss. However, the need to evaluate the FC for exercise prescription becomes a challenge for the obese population (36). To date, obese with comorbidities and morbidly obese individuals are candidates for bariatric surgery and having the possibility to obtain the predicted value of VO_2_ performing a simple and quick functional exercise test in the preoperative period could be very attractive. More importantly, the 2MST performance could also monitor the possible functional loss in the perioperative and postoperative periods, leading to the establishment of adequate rehabilitation strategies for this population.

Our findings are limited to obese with comorbidities and morbidly obese individuals and they cannot be extrapolated to other populations. Thus, we encourage future research to assess the validity of the 2MST for FC evaluation in other disabled populations.

In conclusion, our findings indicated that the 2MST was a viable, practical, and easily accessible way to assess FC in obese with comorbidities and morbidly obese individuals. Our results showed that an exercise test performance (UDS) and BMI can predict peak VO_2_ in these subjects. The introduction of this assessment tool into primary care and hospitals as part of the patients' clinical assessment may allow FC evaluation to become more accessible to morbidly obese patients, facilitating the elaboration of physical exercise prescription and the patients' follow-up.
